# Potential mechanisms and serum biomarkers involved in sex differences in pulmonary arterial hypertension

**DOI:** 10.1097/MD.0000000000019612

**Published:** 2020-03-27

**Authors:** Chan Li, Zeyu Zhang, Qian Xu, Ting Wu, Ruizheng Shi

**Affiliations:** a Department of Cardiovascular Medicine; b Department of Hepatobiliary Surgery; c Department of Cardiovascular Surgery, Xiangya Hospital, Central South University, Changsha, Hunan, China.

**Keywords:** biological markers, pulmonary arterial hypertension, sex characteristics

## Abstract

Pulmonary arterial hypertension (PAH) is a disease associated with high mortality, but notable sex differences have been observed between males and females. For this reason, further research on the mechanisms underlying sex differences in PAH is required to better understand and treat the disease. This study mainly focused on gene expression levels to investigate potential differences in the pathogenesis and progression of PAH between the male and female sexes.

Sex-specific differentially expressed genes (DEGs) were analyzed using the Gene Expression Omnibus datasets GSE117261 and GSE38267. Gene ontology (GO) and Kyoto Encyclopedia of Genes and Genomes (KEGG) pathway analyses were conducted, and a protein-protein interaction (PPI) network was established based on the identified DEGs to predict potential mechanisms involved in the observed sex differences in the pathogenesis of PAH.

We identified 26 female- and 53 male-specific DEGs from lung tissue and 498 female-specific DEGs in blood samples. No male-specific DEGs were identified from blood samples. GO and KEGG pathway analyses revealed that female-specific DEGs in lung tissue were enriched in inflammatory response and cytokine-cytokine receptor interaction, whereas male-specific DEGs were mainly enriched in cellular chemotaxis and the nuclear factor of kappa light polypeptide gene enhancer in B-cell (NF-kappa B) signaling pathway. Lipocalin 2 (LCN2) was the only gene that was differentially expressed in both the lung tissue and the blood of female patients.

In conclusion, inflammation and immunity may play key roles in the pathogenesis of female PAH, and LCN2 may act as a serum biomarker of female PAH, whereas the pathogenesis in males is more complicated.

## Abstract 2

Supplemental Digital Content is available in the text

## 1 Introduction

Pulmonary arterial hypertension (PAH) is a type of pulmonary hypertension (PH) responsible for high mortality and health expenditures^[1]^ and mainly affects small pulmonary arteries, leading to increased pulmonary artery pressure and ultimately right ventricular failure.^[2]^ Unlike other cardiovascular diseases which mainly occur in males, PAH is predominant in females, with a female to male ratio of 2–4:1^[3–5.]^ Males, however, have worse clinical outcomes.^[6]^ This suggests potential differences in the pathogenic mechanisms of PAH among sexes, which should be investigated.

The pathogenesis of PAH is closely associated with gene expression, hormones, oxidative stress, infections, and other factors.^[7,8]^ Immunity and inflammation factors affecting leukocytes or mast cells, such as KIT proto-oncogene (KIT), integrin subunit alpha M (ITGAM), C-X-C motif chemokine receptors (CXCRs), and C-C motif chemokine receptors (CCRs), are reported to play a role as well.^[23–26,34–36]^ Lipocalin 2 (LCN2) is another novel molecular marker whose level was recently reported to be increased in the sera of children with PAH.^[30,31]^

Most studies on PAH did not distinguish among sexes. In those that did focus on differences in mechanisms underlying sex differences, sex hormones have often been regarded as central factors.^[9]^ Unfortunately, the role of estrogen and related pathways in the pathogenesis of PAH is still poorly understood, leading to stagnation in the development of associated therapies.^[10]^ Meanwhile, other mechanisms have been rarely investigated, and effective serum biomarkers are still lacking. Thus, comprehensive analyses of potential mechanisms regarding sex differences in PAH are required.

Bioinformatic analysis is a powerful tool to discover potential molecular markers in the pathology of a disease by analyzing the differential gene expression between patients and healthy controls. It has been widely applied in many fields including cardiovascular medicine.^[11–15]^ To our knowledge, no previous study has used bioinformatic analysis to investigate differences among sexes in the pathogenesis of PAH.

In the present study, expression profiles were obtained from the Gene Expression Omnibus (GEO), and differentially expressed genes (DEGs) between patients with PAH and healthy controls of both sexes were analyzed separately. Further functional analyses were conducted to predict the potential mechanisms involved in the sex difference in the pathogenesis of PAH, and serum biomarkers were also investigated.

## 2 Methods

### 2.1 Data resources

We obtained the raw data for gene expression profiles from the Gene Expression Omnibus (https://www.ncbi.nlm.nih.gov/geoprofiles/),^[16]^ a public repository that provides free access to a full set of microarrays, next-generation sequencing and other forms of high-throughput functional genome data submitted by the scientific community. Only those series with detailed gender metadata were selected in this study. Two series of data (GSE117261 and GSE38267) were included. GSE117261 included expression profiles of lung tissues acquired from 58 PAH patients and 25 healthy controls, which were divided into 2 groups:

(1) a female group with 43 PAH patients and 7 controls,
(2) a male group including 15 PAH patients and 18 controls.

Additionally, the GSE38267 series contained blood samples of 13 PAH patients and 28 controls, with 8 PAH patients and 19 controls in the female group and 5 patients and 9 controls in the male group. The latter series was used to identify potential biomarkers associated with sex differences in PAH (Supplementary Table 1).

Two authors (Chan Li and Zeyu Zhang) independently performed the subsequent analyses, and all results were consistent. Ethics approval for this study was not necessary because the datasets used in this study were obtained from the public database, and the study did not include any patients or volunteers.

### 2.2 Identification of DEGs

We used the R platform (R-project.org) to screen DEGs between PAH patients and healthy controls in the different groups. Fold change (FC) was obtained by calculating the ratio of the expression of each gene between PAH and control. Logarithmic operations with 2, 5, or 10 as base numbers were used to make easier calculations and more scientific comparisons. Genes with |log_2_FC| > 1 were considered as DEGs, and statistical differences were defined by adjusted *P* value < .05. DEGs with log_2_FC < 0 were considered down-regulated, whereas those with log_2_FC > 0 were considered up-regulated. The visualization of DEGs was realized using volcano plots and heat maps using the pheatmap package in R language. Female- and male-specific DEGs were identified by comparing the DEGs between groups.

### 2.3 Gene ontology (GO) and Kyoto encyclopedia of genes and genomes (KEGG) pathway enrichment analysis

To classify identified DEGs according to their functions, GO (a database established by the GO consortium to establish a semantic vocabulary standard applicable to various species) and KEGG (a database of gene pathway information in different species) were used. The gene function classification tool (DAVID, http://david.abcc.ncifc.rf.gov/), an online site for gene annotation and functional enrichment, was used to perform GO analyses to identify potential biological functions of DEGs,^[17]^ and the R package clusterProfiler was used to conduct KEGG pathway enrichment analyses to determine the elevated pathways of the key DEGs.

### 2.4 Protein–protein interaction (PPI) network analysis

STRING (http://string-db.org/), an online database of known protein interactions including both direct physical interactions and indirect functional correlations, was employed to establish a PPI network of DEG-related proteins.^[18]^ Possible hub genes, also known as core regulatory genes which play crucial roles in biological activities, and a core module including a cluster of proteins with the highest degree of correlation in the PPI network were selected and visualized with Cytoscape (V3.5.1; http://cytoscape.org/), a powerful bioinformatic analysis software, with a confidence score > 0.4.^[19]^

## 3 Results

### 3.1 Identification of DEGs based on tissue samples

**Figure 1:**
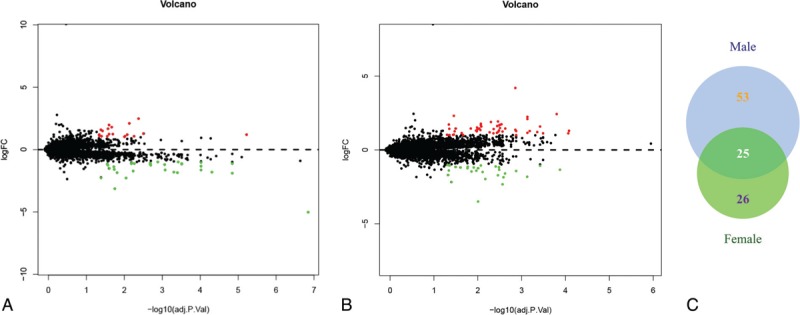
Volcano plot and venn diagram of the differentially expressed genes (DEGs) between normal samples and patients with pulmonary arterial hypertension (PAH) in lung tissues. (A) Volcano plot of DEGs in females. (B) Volcano plot of male-specific DEGs in PAH. Green represents downregulated DEGs; red represents upregulated DEGs; black represents no difference. (C) Venn diagram of DEGs.

Gene expression was calculated according to mapped probes, and an average value was applied if multiple probes matched the same gene. Overall, 20146 genes were analyzed in GSE117261. We found 51 DEGs in female PAH patients compared with those in healthy females, of which 19 were up-regulated and 32 were down-regulated (Fig. 1A). In males, 78 DEGs were identified, including 50 up-regulated and 28 down-regulated genes (Fig. 1B). Interestingly, we defined 26 female-specific DEGs and 53 DEGs exclusive to males, indicating that DEGs may play different roles in the pathogenesis mechanisms of PAH in different sexes (Fig. 1C). The top 15 female- and male-specific DEGs with the highest expression change between patients and controls are listed in Table 1.

**Table 1:**
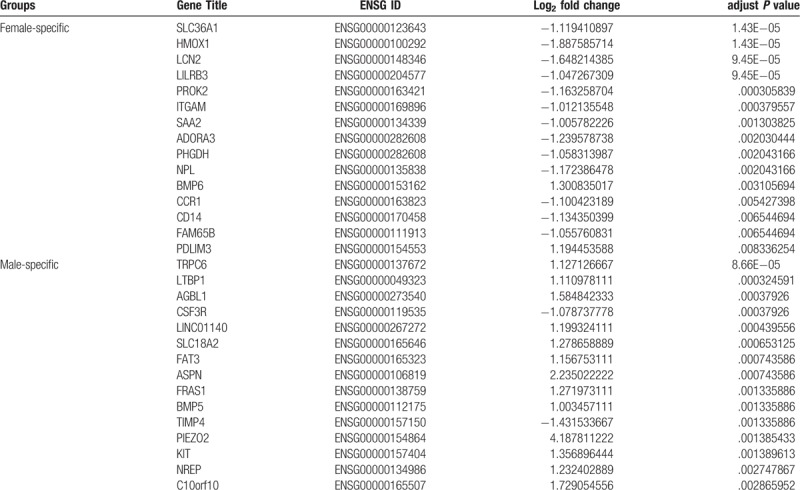
The top 15 female-specific and male-specific differential expressed genes (DEGs) in the lung tissue of patients with pulmonary artery hypertension.

### 3.2 Functional enrichment analysis

**Figure 2:**
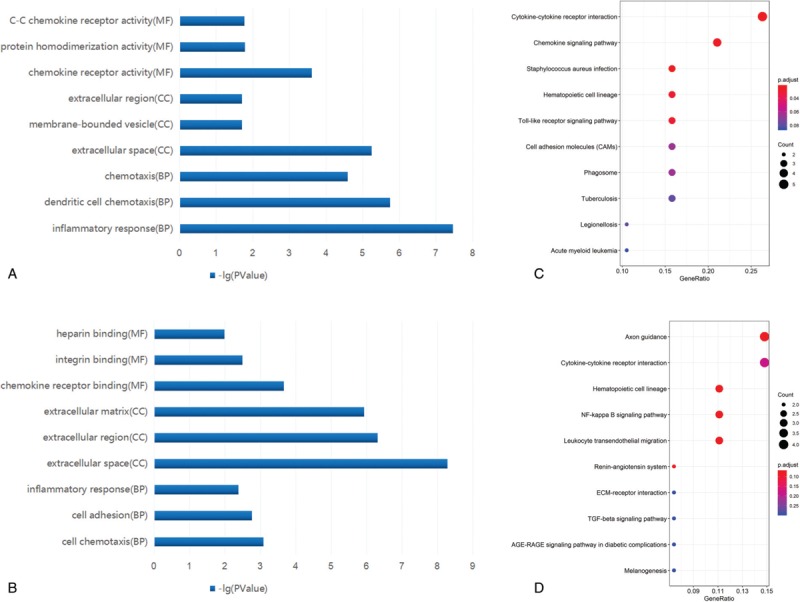
Functional analyses of sex-specific differentially expressed genes (DEGs) in lung tissues. (A) The top 3 enriched Gene Ontology (GO) terms in biological process, cellular component and molecular function of female-specific DEGs. (B) GO enrichment analysis of male-specific DEGs. (C) the enriched Kyoto Encyclopedia of Genes and Genomes (KEGG) pathways in females. (D) the enriched KEGG pathways in males. Dot sizes represent gene number, and dot colors represent the range of adjust *P* values.

To reveal sex-specific GO categories, we performed a functional enrichment analysis of both male- and female-specific DEGs. GO enrichment analysis revealed that female-specific DEGs related to biological processes were mostly enriched in inflammatory response (*P* value < .0001), dendritic cell chemotaxis (*P* value < .0001), and chemotaxis (*P* value < .0001), whereas those relating to cellular components were mainly enriched in extracellular space (*P* value < .0001), membrane-bounded vesicle (*P* value = 0.0190), and the extracellular region (*P* value = .0193). In addition, molecular function analysis showed that female-specific DEGs were involved in chemokine receptor activity (*P* value = .0002), protein homodimerization activity (*P* value = .0159), and C-C chemokine receptor activity (*P* value = .0162) (Fig. 2A).

Male-specific DEGs relating to biological processes were mainly involved in cell chemotaxis (*P* value = .0008), cell adhesion (*P* value = .0017), and inflammatory response (*P* value = .0040). In the cellular component category, male-specific enriched GO terms were associated with extracellular space (*P* value < .0001), extracellular region (*P* value < .0001), and extracellular matrix (*P* value < .0001). Additionally, the molecular function analysis revealed a significant correlation between chemokine receptor binding (*P* value = .0002), integrin binding (*P* value = .0031), heparin binding (*P* value = .0100), and DEGs (Fig. 2B).

To obtain more information about the crucial pathways of these DEGs, a KEGG pathway analysis was performed, and the top 12 significant pathways are shown in Figure 2C and D.

The female-specific DEGs were mostly enriched in cytokine–cytokine receptor interaction (*P* value = .0005), chemokine signaling (*P* value = .0010), and the Toll-like receptor signaling pathway (*P* value = .0019), whereas the male-specific DEGs had a strong correlation with the nuclear factor of kappa light polypeptide gene enhancer in the B-cell (NF-kappa B) signaling pathway (*P* value = .0047), cytokine–cytokine receptor interaction (*P* value = .0171), the transforming growth factor-beta (TGF-beta) signaling pathway (*P* value = .0410), and the advanced glycosylation end-product-receptor for advanced glycosylation end-product (AGE-RAGE) signaling pathway in diabetic complications (*P* value = .0458).

### 3.3 PPI network analysis

**Figure 3:**
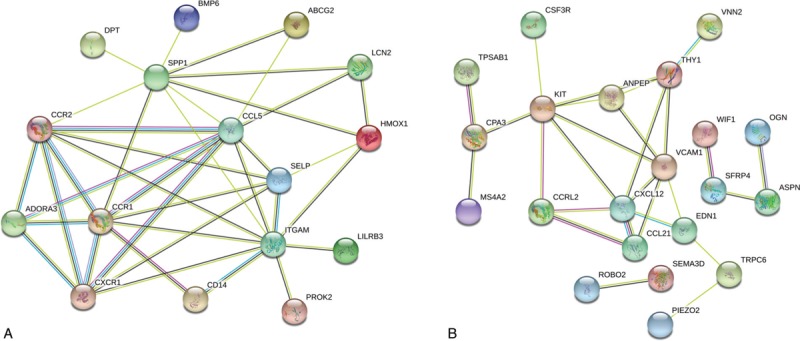
Protein–protein interaction (PPI) network analysis in lung tissues. PPI network of (A) female-specific DEGs and (B) male-specific DEGs.

Using the STRING database, we performed PPI network analysis for female- and male-specific DEGs. As shown in Figure 3, 16 nodes were identified in females, and 21 in males. ITGAM, C-C motif chemokine ligand 5 (CCL5), secreted phosphoprotein 1 (SPP1), CCR1, CCR2, CXCR1, selectin P (SELP), adenosine A3 receptor (ADORA3), heme oxygenase 1 (HMOX1), and LCN2 were the top 10 hub genes in females. In males, KIT, C-X-C motif chemokine ligand 12 (CXCL12), vascular cell adhesion molecule 1 (VCAM1), Thy-1 cell surface antigen (THY1), alanyl aminopeptidase (ANPEP), CCL21, C-C motif chemokine receptor like 2 (CCRL2), carboxypeptidase A3 (CPA3), endothelin 1 (EDN1) and asporin (ASPN) had the most abundant connectivity (Table 2).

**Table 2:**
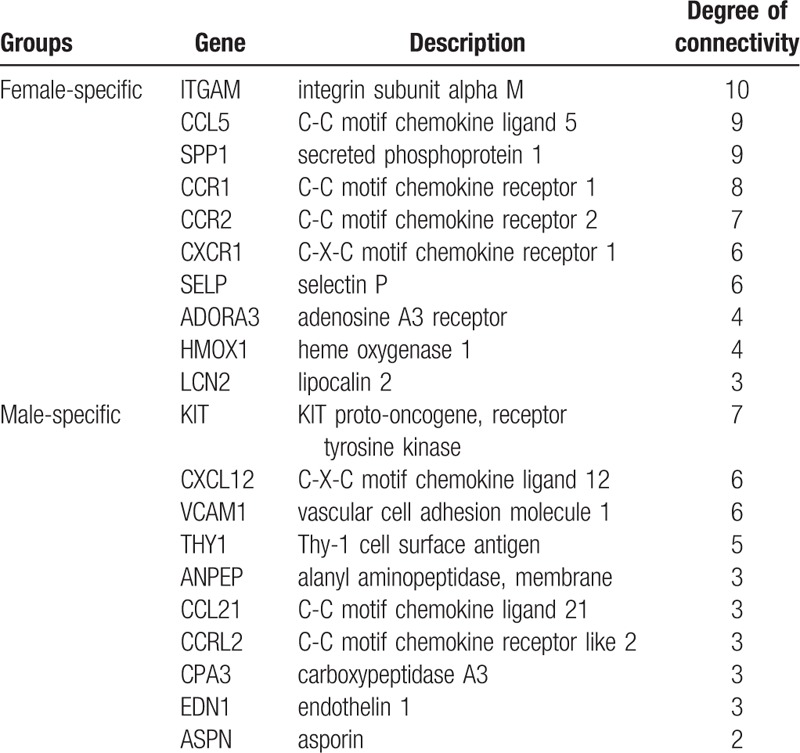
The top 10 hub genes identified in protein-protein interaction (PPI) network in lung tissue.

### 3.4 Identification of DEGs based on blood samples

**Figure 4:**
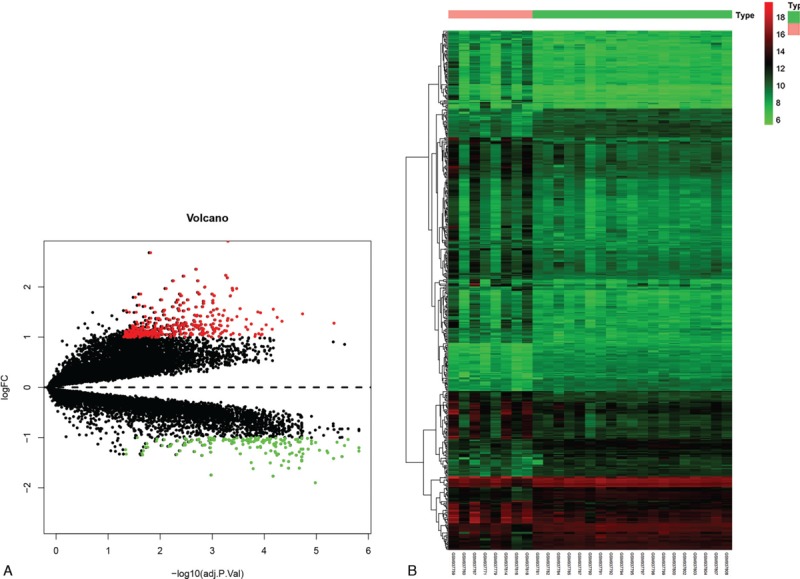
Volcano plot and heat map of the differentially expressed genes (DEGs) in the blood of female PAH compared with normal samples. (A) Volcano plot of DEGs in females. Green represents downregulated DEGs; red represents upregulated DEGs; black represents no difference. (B) Heat map of DEGs. Red represents greater expression and green represents less expression.

To identify DEGs in blood, we analyzed the GSE38267 series of blood samples, including 17227 genes. Interestingly, 498 DEGs were found in female blood samples between PAH patients and controls (Fig. 4A and 4B), whereas no DEGs were found in male blood samples (Supplementary Fig. 1). Functional analyses were also conducted, and the results are shown in Supplementary Figure 2 and 3.

### 3.5 Identification of potential serum biomarkers in female PAH

To further investigate potential serum biomarkers related to PAH pathogenesis, we compared the results from both the lung tissue and blood analysis, and LCN2 was the only gene that was differentially expressed both in the blood and the lungs of female PAH patients.

## 4 Discussion

PAH is a lethal disease that still lacks effective methods for prevention, diagnosis, and treatment. Since notable sex differences have been recognized in PAH,^[20]^ a better understanding of potential mechanisms underlying the sex differences in the pathogenesis and progression of PAH may aid future studies. In the present study, we investigated the gene expression profile of lung tissues and blood samples in patients with PAH. By comparing these data with those of healthy people, DEGs were identified and functional analyses were performed in both males and females. Our results initially revealed differences in the pathogenesis between sexes in PAH, and LCN2 was identified as a serum biomarker for female PAH, providing a basis for further research and possible sex-specific diagnosis.

GO and KEGG pathway analyses showed that female-specific DEGs were up-regulated in biological processes related to inflammation and immunity, which corroborates prior studies.^[7,21]^ Interestingly, pathways involving sex hormones which had been previously recognized as important in PAH pathogenesis, such as the serotonin signaling system,^[22]^ were not found to be enriched in functional enrichment analyses of female patients, indicating these pathways could be either a common pathogenesis factor in both sexes, or statistically insignificant in this study because of the relatively small cohort. Furthermore, by conducting PPI network analyses, we also identified a few hub genes relating to female-specific DEGs. Most of these hub genes were related to immunity or inflammation, which is consistent with the functional analyses results, and agrees with previous studies of PAH pathogenesis, which generally did not distinguish among sexes. For instance, ITGAM, also known as CD11b, is a marker of leukocytes including neutrophils, monocytes, macrophages, and NK cells, and is closely related to inflammation in PAH.^[23,24]^ CXCRs and CCRs both encode chemokine receptors which, in combination with CCLs, are critical for the recruitment of immune cells to the site of inflammation.^[25,26]^ Further studies focusing on female patients are warranted, and potential therapies targeting these factors may be useful for the prevention and treatment of PAH, especially in females.

LCN2, also known as neutrophil gelatinase-associated lipocalin, encodes a protein of the lipocalin family which is secreted by various cell types and implicated in apoptosis, innate immunity, and the development of kidney disease and heart failure.^[27–29]^ Recent studies have partially revealed its significant role in PAH. Serum LCN2 level was increased in children with PH in two different studies.^[30,31]^ However, these studies did not distinguish sexes, and only children were included, limiting their ability to illuminate the role of LCN2 in different ages and different sexes. In cultured human pulmonary arterial smooth muscle cells, it was demonstrated that LCN2 significantly promoted endoplasmic reticulum (ER) stress by increasing intracellular iron levels^[32]^ and activating phosphatidylinositol 3-kinase/AKT serine/threonine kinase (PI3K/Akt) pathway to promote proliferation,^[33]^ which has been proposed to be essential for vascular remodeling in PH. In this study, LCN2 was the only gene differentially expressed both in the blood and the lungs of females. It was also predicted to be a hub gene in the PPI analysis regarding lung tissue, which emphasized its role in the pathogenesis of PAH in females instead of in males. More studies are required to investigate the mechanisms of LCN2 and their effect on female PAH patients. Furthermore, whether LCN2 is capable of acting as a serum biomarker or guiding treatment of PAH in female is another valuable topic for future studies.

As for males, it was shown that male-specific hub genes were involved in more aspects other than the chemokine and leukocyte pathways which were also enriched in females. For instance, KIT (CD117) is a gene encoding proto-oncogene c-kit, which is a transmembrane receptor for mast cell growth factor, and it may participate in vascular remodeling in PAH.^[34–36]^ In addition, CPA3 encodes a member of the carboxypeptidase A family of zinc metalloproteases and preproprotein which is released by mast cells, and may be involved in the degradation of endogenous proteins in PAH.^[37]^ Taken together, these results suggest that the mast cells may play a crucial role in male PAH.

There were several limitations in our study. Firstly, only two gene series could be used because the others in the GEO database were not classified by patient gender. This reduced the number of samples available for inclusion, reducing the sample size. Secondly, microarray analysis could not directly demonstrate protein expression. Polymerase chain reaction (PCR) or western blot analysis should be done to confirm our results. Further studies should be carried out to understand the exact mechanisms of sex-specific PAH in vivo or in vitro. Clinical studies would also be helpful to further validate our results.

## 5 Conclusion

Taken together, our study identified sex-specific DEGs between PAH and control samples from lung tissues and blood. Our results suggest that inflammation and immunity may play essential roles in the pathogenesis of female PAH, and LCN2 may serve as a serum biomarker for females, whereas pathogenesis in males is more complicated. Further studies, including experimental validation of the hub genes and serum biomarker (LCN2), should be carried out to verify our results.

## Author contributions

All authors made substantive intellectual contributions to this study to qualify as authors. Ruizheng Shi conceived of the design of the study. Chan Li, Zeyu Zhang performed the study, collected the data, and contributed to the design of the study. Chan Li, Zeyu Zhang drafted the manuscript. Qian Xu, Ting Wu revised the manuscript. All authors read and approved the final manuscript.

## Supplementary Material

Supplemental Digital Content

## Supplementary Material

Supplemental Digital Content

## Supplementary Material

Supplemental Digital Content

## Supplementary Material

Supplemental Digital Content
