# Supplementary material for: Potential mechanisms and serum biomarkers involved in sex differences in pulmonary arterial hypertension
Source: Medicine (Baltimore). 2020 Mar 27;99(13):e19612. doi: 10.1097/MD.0000000000019612 (PMC7220321; doi:10.1097/MD.0000000000019612)

Supplement Figure 1: Volcano plot of the differentially expressed genes (DEGs) in the blood of male PAH compared with normal samples. Black represents no difference


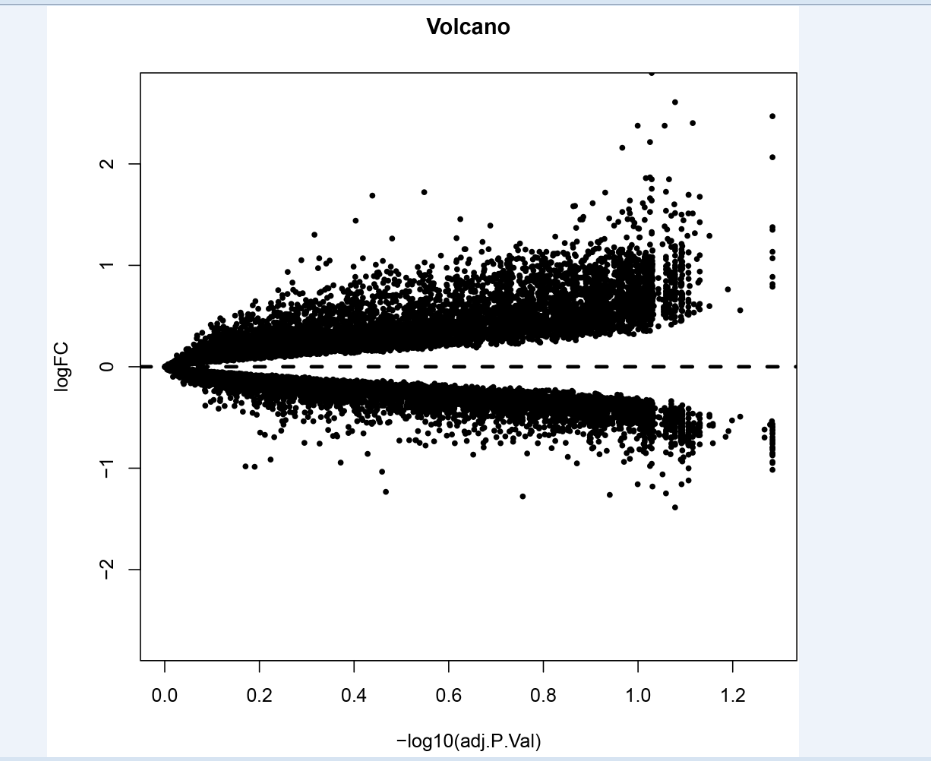

Supplement: Supplemental Digital Content [file medi-99-e19612-s001.doc]
