# Supplementary material for: Potential mechanisms and serum biomarkers involved in sex differences in pulmonary arterial hypertension
Source: Medicine (Baltimore). 2020 Mar 27;99(13):e19612. doi: 10.1097/MD.0000000000019612 (PMC7220321; doi:10.1097/MD.0000000000019612)

Supplement Figure 2: Functional analyses and Protein-protein interaction (PPI) network analysis of differentially expressed genes (DEGs）in the blood of female PAH patients. (a) the top 3 enriched Gene Ontology (GO) terms in biological process, cellular component and molecular function of female DEGs. (b) the enriched Kyoto Encyclopedia of Genes and Genomes (KEGG) pathways.


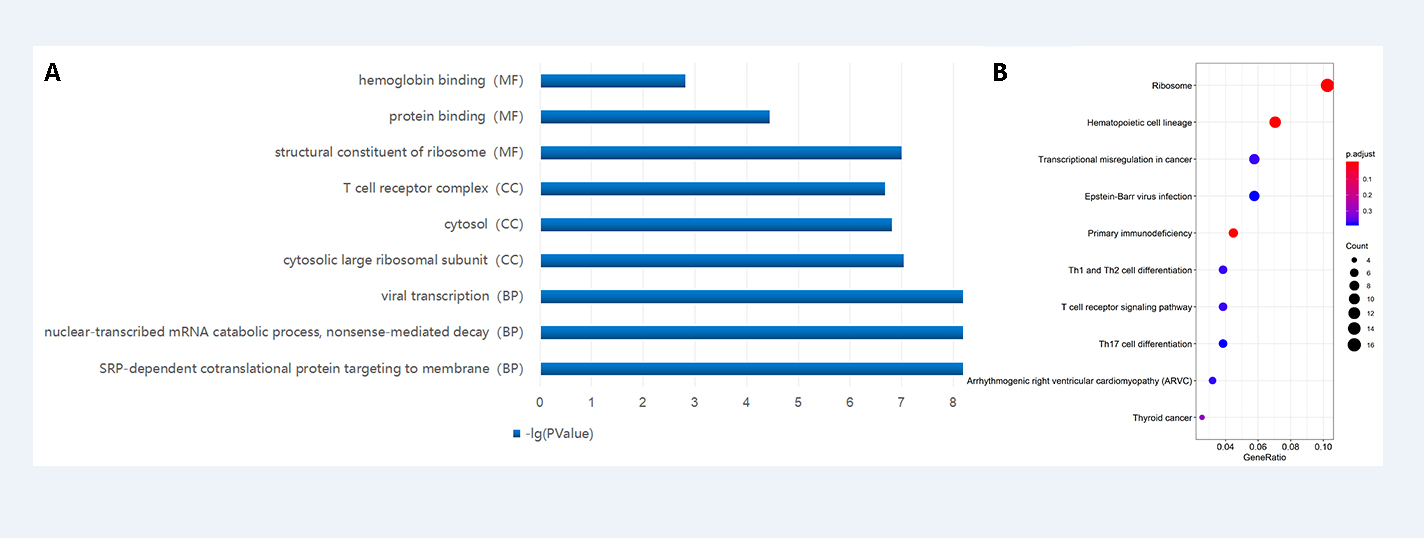

Supplement: Supplemental Digital Content [file medi-99-e19612-s002.doc]
