# Supplementary material for: Potential mechanisms and serum biomarkers involved in sex differences in pulmonary arterial hypertension
Source: Medicine (Baltimore). 2020 Mar 27;99(13):e19612. doi: 10.1097/MD.0000000000019612 (PMC7220321; doi:10.1097/MD.0000000000019612)

Supplement Figure 3: Protein–protein interaction (PPI) network of female DEGs identified from the blood samples.


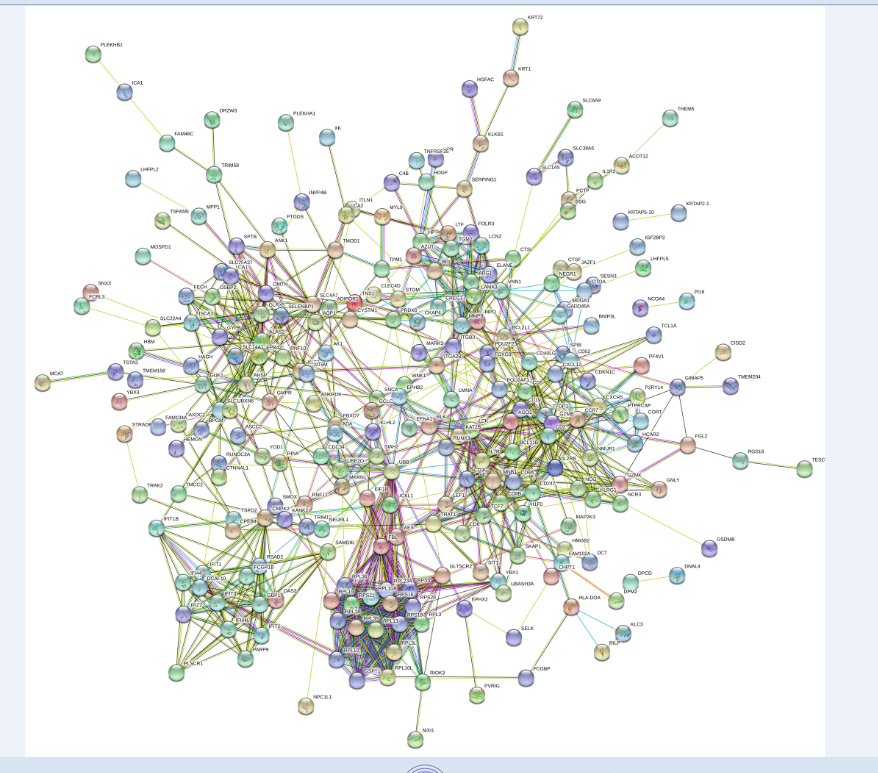

Supplement: Supplemental Digital Content [file medi-99-e19612-s003.doc]
