# Supplementary material for: Potential mechanisms and serum biomarkers involved in sex differences in pulmonary arterial hypertension
Source: Medicine (Baltimore). 2020 Mar 27;99(13):e19612. doi: 10.1097/MD.0000000000019612 (PMC7220321; doi:10.1097/MD.0000000000019612)
Supplement: Supplemental Digital Content [file medi-99-e19612-s004.doc]

**Supplement Table 1: Information on platform and number of samples in each series.**

| Gene Expression Omnibus ID | Gender | PAH sample count | Control sample count | Platforms |
| --- | --- | --- | --- | --- |
| GSE117261 | female | 43 | 7 | Affymetrix Human Gene 1.0 ST Array |
|  | male | 15 | 18 |  |
| GSE38267 | female | 8 | 19 | Agilent-028004 SurePrint G3 Human GE 8x60K Microarray |
|  | male | 5 | 9 |  |
